# Fourier Transform Analysis of GPS-Derived Mobility Patterns for Diagnosis and Mood Monitoring of Bipolar and Major Depressive Disorders: Prospective Study

**DOI:** 10.2196/71658

**Published:** 2025-07-15

**Authors:** Ting-Yi Lee, Ching-Hsuan Chen, Chih-Min Liu, I-Ming Chen, Hsi-Chung Chen, Shu-I Wu, Chuhsing Kate Hsiao, Po-Hsiu Kuo

**Affiliations:** 1 Institute of Epidemiology and Preventive Medicine, College of Public Health, National Taiwan University, Room 521, No. 17, Xuzhou Road, Taipei, 10055, Taiwan, 886 2 33668015; 2 Department of Obstetrics and Gynecology, Taipei City Hospital Heping Fuyou Branch, Taipei, Taiwan; 3 Department of Psychiatry, National Taiwan University Hospital, Taipei, Taiwan; 4 Department of Psychiatry, Center of Sleep Disorders, National Taiwan University Hospital, Taipei, Taiwan; 5 College of Medicine, National Taiwan University, Taipei, Taiwan; 6 Department of Psychiatry, Mackay Memorial Hospital, Taipei, Taiwan; 7 Department of Medicine, MacKay Medical College, New Taipei City, Taiwan; 8 Institute of Health Data Analytics and Statistics, College of Public Health, National Taiwan University, Taipei, Taiwan; 9 Department of Public Health, College of Public Health, National Taiwan University, Taipei, Taiwan; 10 Psychiatric Research Center, Wan Fang Hospital, Taipei, Taiwan

**Keywords:** ecological momentary assessment, digital phenotyping, mobility, mood disorder, major depressive disorder, bipolar disorder

## Abstract

**Background:**

Mood disorders, including bipolar disorder (BP) and major depressive disorder (MDD), are characterized by significant psychological and behavioral fluctuations, with mobility patterns serving as potential markers of emotional states.

**Objective:**

This study explores the diagnostic and monitoring capabilities of Fourier transform, a frequency-domain analysis method, in mood disorders by leveraging GPS data as an objective measure.

**Methods:**

A total of 62 participants (BP: n=20, MDD: n=27, and healthy controls: n=15) contributed 5177 person-days of data over observation periods ranging from 5 days to 6 months. Key GPS indicators—location variance (LV), transition time (TT), and entropy—were identified as reflective of mood fluctuations and diagnostic differences between BP and MDD.

**Results:**

Fourier transform analysis revealed that the maximum power spectra of LV and entropy differed significantly between BP and MDD groups, with patients with BP exhibiting greater periodicity and intensity in mobility patterns. Notably, participants with BP demonstrated consistent periodic waves (eg, 1-d, 4-d, and 9-d cycles), while such patterns were absent in those with MDD. In addition, after adjusting for age, gender, and employment status, only the power spectrum of LV remained a significant predictor of depressed mood (odds ratio [OR] 0.9976, 95% CI 0.9956‐0.9996; *P*=.02). Daily GPS data showed stronger correlations with ecological momentary assessment (EMA)–reported mood states compared to weekly or monthly aggregations, emphasizing the importance of day-to-day monitoring. Depressive states were associated with reduced LV (OR 0.975, 95% CI 0.957‐0.993; *P*=.008) and TT (OR 0.048, 95% CI 0.012‐0.200; *P*<.001) on weekdays, and lower entropy (OR 0.662, 95% CI 0.520‐0.842; *P*=.001) on weekends, indicating that mobility features vary with social and temporal contexts.

**Conclusions:**

This study underscores the potential of GPS-derived mobility data, analyzed through Fourier transform, as a noninvasive and real-time diagnostic and monitoring tool for mood disorders. The findings suggest that the intensity of mobility patterns, rather than their frequency, may better differentiate BP from MDD. Integrating GPS data with EMAs could enhance the precision of clinical assessments, provide early warnings for mood episodes, and support personalized interventions, ultimately improving mental health outcomes. This approach represents a promising step toward digital phenotyping and advanced mental health monitoring strategies.

## Introduction

Among global health priorities, mood disorders, particularly major depressive disorder (MDD), and bipolar disorder (BP), have gained increasing attention. Clinical diagnosis and treatment for these disorders traditionally rely on patient visits, which often provide only sporadic data, making it challenging for clinicians to fully grasp the patient’s condition [1]. Factors such as cognitive impairment and age can further affect the reliability of subjective measurements. This underscores the potential of digital phenotyping tools for objective measurement. In Taiwan, smartphone ownership has reached nearly 90%, presenting an opportunity to use mobile devices as digital mental health tools in recent years. GPS technology, specifically, allows for the passive tracking of individuals’ mobility behaviors, offering a novel means to identify psychological disorders [2]. GPS-based monitoring captures clinical symptoms by analyzing movement patterns across various locations [3]. These trajectories can provide insights into behavioral patterns, identifying primary locations such as home, work, or social venues, thereby gaining insights into patients’ behavioral patterns [4].

While subjective questionnaires such as the Hamilton Rating Scale for Depression (HAM-D), Beck Depression Inventory (BDI), Patient Health Questionnaire-4 (PHQ-4), and Beck Anxiety Inventory (BAI) are widely used, they only capture average emotional states over 1 to 2 weeks. This retrospective approach introduces temporal biases and overlooks daily emotional fluctuations [1]. Studies have shown correlations between increased mobility as captured by GPS and reduced depressive symptoms [5], suggesting that GPS-derived features such as location variance (LV), speed mean (SM), transition time (TT), and entropy can reflect emotional states [6]. Entropy-related variables showed the strongest correlation with depression. Within the distance dimension, LV demonstrated a significant negative correlation with depression [7]. However, there remains a lack of clarity regarding which time frames and GPS indicators are most relevant for emotional symptom evaluation.

Understanding the differences in GPS indicators between patients with BP and MDD is crucial. While many studies have examined the relationship between GPS indices and depression status—focusing on either BP [8] or MDD [9] —most have relied on time-domain analyses of long-term GPS data [10-12]. However, fewer studies have explored the potential of frequency-domain methods, such as Fourier transforms or wavelet analyses. Frequency-domain techniques excel in characterizing periodic mobility patterns and offer superior discriminative power for identifying distinct features of mobility [13]. For instance, comparative analyses of time, frequency, and wavelet features across various activities have demonstrated that frequency-domain features generally outperform others [14]. Past research has mostly focused on frequency domain analysis of human activity recognition or physiological signal analysis, such as gait analysis, and motion pattern recognition, including wavelet analysis, Fourier transform, and power spectral density. Wavelet transform excels in extracting time-frequency features, particularly for nonstationary signals like speech and EEG, offering enhanced performance through multiresolution analysis [15], while power spectral density is widely used in activity recognition (eg, distinguish fall events from other intense activities) and heart rate variability analysis [16]. On the other hand, Fourier transform is ideal for analyzing steady-state and periodic activities, showing superior classification accuracy, especially in noisy conditions [17]. This highlights Fourier transform as a robust method for characterizing short-duration stationary signals, with proven efficacy in distinguishing physical activities like level walking, stair ascent, and stair descent. Despite its advantages, the application of frequency-domain methods to reflect participants’ mobility periodicity and stability remains limited in current research.

Ecological momentary assessment (EMA) provides a robust alternative for longitudinal and continuous mood monitoring. EMA involves real-time, repeated self-reports of experiences, enabling the tracking of mood fluctuations with high temporal resolution [18]. Both patients with BP and MDD, even during euthymic states, may experience residual symptoms, which can lead to emotional instability even in the absence of major episodes. Interestingly, GPS-derived mobility patterns show a stronger relationship with depressive symptoms on weekends compared to weekdays [19]. This distinction may stem from the influence of social roles and expectations, which significantly shape mobility patterns. For instance, working professionals and students typically commute to work or school on weekdays, establishing predictable routines. However, on weekends, individuals may exhibit reduced mobility, particularly if they feel down or experience depressive symptoms, highlighting the importance of considering temporal and social contexts in understanding mood-related changes in mobility.

Integrating EMA with GPS mobility patterns could enable detailed insights into short-term mood changes, particularly for BP and MDD, which share depressive features but differ in clinical presentation. For example, BP includes manic episodes, whereas MDD does not. Patients with BP may exhibit more frequent mobility fluctuations compared to patients with MDD. Research has highlighted the potential of GPS patterns in predicting daily depressive states, with features such as LV, TT, and entropy showing greater relevance on specific days [1].

This study aims to evaluate the relationship between GPS mobility indicators, clinical symptoms, and EMA-reported mood states using Fourier transform analysis. We hypothesize that patients with BP may exhibit more frequent mobility fluctuations compared to patients with MDD, and the maximum power spectrum is more effective than the frequency period in distinguishing between BP and MDD diagnoses, providing valuable insights for earlier diagnosis and intervention. In addition, we will explore the impact of varying time spans on the accuracy and relevance of GPS-based emotional assessments.

## Methods

### Participants

**Figure 1.**
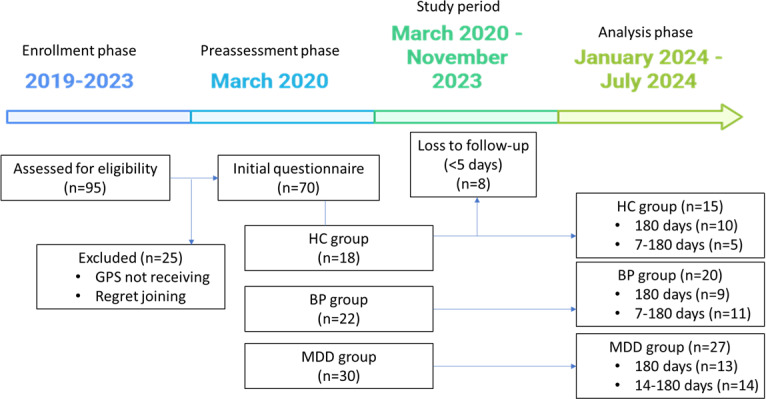
Flow chart of the study period. BP: bipolar disorder; HC: healthy control; MDD: major depressive disorder.

We conducted a prospective study over a 6-month period to explore GPS-derived mobility patterns as markers using Fourier transform for frequency-domain analysis to differentiate diagnosis for mood disorders. The study recruited a sample of 62 participants, comprising healthy control (HC; n=15), BP (n=20), and MDD (n=27; see Figure 1). Patients were recruited from the psychiatric departments of 3 hospitals (National Taiwan University Hospital, Mackay Memorial Hospital, and Taiwan Adventist Hospital) in Taipei, Taiwan, targeting individuals diagnosed with MDD and BP. Patients were recruited through clinical referrals from psychiatrists in both outpatient and inpatient settings, ensuring accurate diagnostic information. The sample comprised patients referred consecutively from outpatient clinics who attended routine follow-up visits and those in partial remission from inpatient units from March 2020 to November 2023. HC were recruited from the same hospital catchment areas by inviting well individuals (visitors and passersby) to participate. HC were screened for the absence of psychiatric disorders using standardized diagnostic interviews. All participants underwent structured clinical interviews conducted by trained research staff to confirm eligibility and gather additional data. Inclusion criteria for patients were as follows: (1) confirmation of MDD or BP according to the *Diagnostic and Statistical Manual of Mental Disorders, Fifth Edition,* criteria, aged between 20 and 65; (2) absence of substance-induced mood disorders, intellectual impairment, and schizophrenia; (3) experienced at least 2 episodes of depressive or manic episodes from the interview questionnaires using the modified Chinese version of the Schedule for Affective Disorders and Schizophrenia-Lifetime (SADS-L) [20] to ensure the validity of the diagnosis, as previously recommended by [21]; (4) Young Mania Rating Scale (YMRS) scores <16 and HAM-D scores <16, ensuring that they were not in an acute phase of illness [22,23]. This increases the likelihood of stable follow-up over 6 months. In addition, patients in an acute state who require hospitalization in Taiwan are typically not allowed to use mobile phones, meaning their motility patterns would not reflect the daily life of outpatients or individuals in a more stable condition. In addition, the inclusion criteria for HC were: (1) general population aged between 20 and 65, (2) no history of psychiatric disorders or intellectual developmental disorders, and (3) non-night shift workers.

### Ethical Considerations

This study was approved by the institutional review boards of National Taiwan University Hospital (IRB 201811087RINA), and all patients gave written informed consent. The GPS data collected via mobile devices were encrypted at the collection point, ensuring that the recorded data did not contain the original latitude and longitude coordinates. Furthermore, the GPS data were inaccessible on the device itself, and additional encryption protocols safeguarded the data during transmission. The decryption of the data was only possible upon reaching the researchers, at which point the original GPS coordinates could be retrieved. Participants received a monetary compensation of approximately US $22 after completion of the EMA survey answers.

### Beiwe Data Acquisition

We utilized the Beiwe application on each participant’s smartphone for 6 months to conduct GPS surveys. The follow-up period for this study lasted 6 months, during which passive data (eg, GPS data) and active data (eg, daily mood EMA to assess emotional fluctuations) were collected through the Beiwe app management platform. During the follow-up period, some participants withdrew from the study and provided only limited recorded data. Ultimately, data of 62 participants, totaling 6330 person-days, were included in the final analysis. Beiwe is a mobile app developed by the Onnela team at Harvard University [24]. Through collaboration, we designed the Chinese version of the software (see Figure S1 in Multimedia Appendix 1) to record participants’ objective data, facilitating dynamic data collection. The goal of collecting this data is to contribute to clinical research understanding the correlation between emotional changes and various mobility patterns in daily life. Regarding the recording of mobility ranges, we employed anonymized information using map coordinates as records. This approach ensures that sensitive information such as location types or the number of floors entered and exited is not disclosed, thereby preserving individual privacy. The confidentiality of relevant information is strictly maintained in accordance with the terms outlined in the informed consent. These data are solely intended for academic research purposes and will not be used for any commercial activities, emphasizing the confidentiality and security of the information collected. Implementation challenges for studies of this nature include 3 main aspects: data feature extraction, data granularity, and data sparsity, encompassing issues of data accuracy, sparsity, and compliance [5].

In our study, data feature extraction refers to how meaningful indicators such as GPS raw data are transformed into mobility patterns. We aim for these meaningful indicators to accurately reflect potential clinical states. Here, we adopt the definitions and formulas of GPS features established by a previous study [25], LV, SM, speed variance (SV), number of clusters (NC), entropy, homestay, TT, and total distance (TD). We analyzed these GPS-derived mobility metrics to assess participants’ movement patterns. LV measures the variability in a participant’s GPS location, while SM and SV capture the average and variability of instantaneous speed at each GPS data point, respectively. NC identifies the key locations where participants spent most of their time, and homestay represents the percentage of time spent at home. TT reflects the proportion of time spent moving between locations, whereas TD is calculated as the sum of the Euclidean distances between consecutive location points. Finally, entropy quantifies how each participant’s time was distributed across different location clusters [25].

The data granularity in this study pertains to the frequency of GPS data collection. In practice, GPS is the most power-consuming sensor. To minimize battery consumption, the Beiwe platform records GPS data using a cyclic sampling approach. Specifically, GPS data are collected for one minute during each ’on-cycle’ (active period), followed by a ten-minute ’off-cycle’ (inactive period) during which no GPS data are recorded [26].

In terms of data sparsity and compliance, persistence involves compliance, specifically addressing the issue of missing values. During the follow-up period, participants were requested to enable location services on their smartphones and fill out daily emotional state surveys within the app. These surveys include questionnaires designed to measure emotional well-being, contributing to the completeness of the dataset.

### GPS Data Processing

Data loss occurred due to various reasons, including equipment malfunction, technical issues with device usage, such as airplane mode, software errors, participants forgetting to charge their phones or devices, server and network connection problems, sensor damage, lack of clinical data for comparison with sensor data, and disruptions caused by mobile software updates [2]. The Beiwe platform typically activates GPS data collection at a frequency of 1 Hz within a one-minute on-cycle and then pauses data collection for ten minutes during the off-cycle. Ideally, there should be around 327 data points per hour (60 min ÷ 11 min per cycle × 60 s). Figure S2 in Multimedia Appendix 1 illustrates the data distribution for Participant M01 over 26 weeks. In the case of M01, the participant with a relatively high volume of GPS data can collect as many as 300 data points per hour. If the obtained data are less than expected, several factors might explain this discrepancy, including (1) GPS deactivation: the participant might have intentionally turned off the GPS function on their device; (2) partial GPS sensor response: the GPS sensor on the participant’s phone might only respond to a portion of location data queries; (3) insufficient sampling duration: the GPS sampling duration might be less than the required time; or (4) mismatched sampling frequency: the GPS sampling frequency might differ from the specified frequency. This can happen if participants use different phone models or devices from various manufacturers, leading to variations in GPS sensor data [27]. The mobile phone brands used by participants in this study included iPhone, ASUS, Samsung, Xiaomi, HTC, OPPO, HUAWEI, SONY, VIVO, and NOKIA. Due to variations in phone models and manufacturers, there may be differences in GPS sensing and sensor data accuracy among the devices.

For location data, the data collection process followed the protocol established by the Onnela Lab research [26]. Preprocessing defines stationary and moving to avoid GPS errors and for subsequent analysis. First, exclude data with GPS errors exceeding 50 meters. Subsequently, calculate the average position over a 10-second interval to mitigate outliers, considering positions with no data for over 10 seconds as missing values (NA). Check the distance from the next data; if it exceeds √ 10 meters measured in 10 seconds, it is considered as movement. If the next data are considered NA, it is considered unknown, and it is defined that a maximum displacement of 75 meters or more within 6 minutes is considered as a movement to confirm the “stationary state” or “moving state.” GPS features in the stationary state: LV, NC, entropy, and homestay. The GPS feature in the moving state: TT. GPS features that do not distinguish between moving or stationary states: TD, SM, and SV. The suggestion of the Onnela lab team [26] is that for smartphone GPS data with severe missing degrees, more careful processing is needed, using data from other days to fill in these distances and using imputation methods (see Figure S3 in Multimedia Appendix 1). In brief, we applied linear interpolation for trajectory imputation and inferred the GPS features under 10 times simulation. This method allowed us to achieve a more accurate representation of the data by filling in gaps and reducing noise, ultimately leading to improved performance in our predictive models.

### Fourier Transform Analysis for Mobility Patterns

The data collected by GPS are in the time domain. Fourier transform is a method that converts time-domain signals into frequency-domain signals. The signal refers to the same entity, whether represented in the time domain (waveform) or the frequency domain (spectrum). The data were transformed into sine and cosine waveforms when entering the data analysis phase. These waveforms are then superimposed to depict the frequency cycles of motion. This analysis obtained two primary variables: the power spectrum and the frequency. Complex waveforms can be segmented to separate various frequency components. After performing the Fourier transform, we derived two main variables: frequency, which represents the mobility patterns’ periodicity, and the power spectrum for each frequency, which indicates the intensity of these mobility patterns.

### Clinical Questionnaire Measurement and Ecological Momentary Assessment

The severity of the disease was assessed using the HAMD-17 [28] for depressive episodes and the YMRS [29] for manic episodes. Symptom severity was further evaluated with the BDI-II [30], which evaluates the severity of current depression over a two-week period. The BDI-II critical scores are 13, 19, and 28, corresponding to mild, moderate, and severe depression, respectively. Anxiety levels were measured using the BAI [31], which evaluates symptoms over the past week. The BAI cutoff scores are 9, 16, and 29, indicating mild, moderate, and severe anxiety. In addition, the PHQ-4 [32], a brief self-report, was used to screen for depression and anxiety. Two items are used as screeners for depression and two items for anxiety. Participants completed daily EMA questionnaires per day via the Beiwe app throughout the study period. The EMA captured self-reported states of depression, mania, fatigue, and irritability, with response options ranging from 1 (not at all) to 5 (seriously); see Table S1 in Multimedia Appendix 1. The EMA mood items were adapted from validated questions in published studies on treatment response, ensuring consistency and reliability [33]. Our EMA data were collected daily at 12:45 PM, and if there were missing data, imputation was performed using the EMA collected at 5:00 PM. With twice-daily reports, the intraclass correlation coefficients for mood states were as follows: 0.888 for manic mood, 0.999 for irritability, 0.920 for depressive mood, and 0.747 for fatigue [34].

### Statistical Analysis

Our study was based on prospective data collection. The exposures are digital phenotyping GPS features, and the outcome was daily depression score or diagnosis. Age, gender, and employment status were considered potential confounding factors and were adjusted for in models. The sample size was estimated based on previously reported correlations between GPS features and mood-related symptoms [25], which ranged from 0.49 to 0.63. Assuming a conservative correlation coefficient of *r*=0.5, an α level of .05, and desired power levels of .90, the required sample sizes were 38 [34]. The Kruskal-Wallis test was used to determine whether the medians of multiple independent groups, which do not follow a normal distribution, were equal. To gain a comprehensive understanding of the relationship between GPS features and clinical status, polyserial correlations were calculated for continuous and ordinal variables, identifying patterns within and between BP, MDD, and HC groups. In addition, Pearson correlation coefficients and *P* values were computed to assess linear relationships. A logistic regression model was used to evaluate the ability of GPS features to differentiate between affective and euthymic statuses. For comparisons between BP and MDD groups, the Mann-Whitney *U* test was applied to examine differences when the dependent variable was either ordinal or a non-normally distributed continuous variable. In addition, we used QGIS 3.14 software (QGIS Development Team) to overlay Google Maps layers for map visualization.

## Results

### Demographic and Clinical Variables

The demographic and clinical characteristics of the study participants are presented in Table 1. Due to variance heterogeneity, the Kruskal-Wallis test was used to compare differences among groups. No significant differences were observed in sex, age, or occupation. Both BP and MDD groups exhibited higher BDI (*P*<.001), BAI (*P*<.001), and PHQ-4 (*P*<.001) scores compared to the HC group. Patients’ BDI scores were used to indicate their depressive severity during the study period. It was found that 23.4% (11/47) of patients experienced moderate depression, while 25.5% (12/47) were in severe depression, suggesting a noticeable level of mood fluctuation. Patients with BP and MDD exhibited substantially greater instability: we found that depression mood fluctuation occurred on 44% (1577/3584) of days in patients with MDD and BP compared to 9% (103/1148) in controls, while manic mood fluctuation was observed on 48% (716/1504) of days in patients with BP versus 8% (94/1148) in controls. These findings demonstrate that HC maintain stable mood trajectories, whereas patients with BP and MDD experience marked oscillations, which underscores mood instability as a transdiagnostic clinical marker. Although the exact number of individuals who experienced a relapse cannot be determined, 48.9% (23/47) exhibited moderate to severe depression. While the BDI is not a tool for assessing the acute phase, it serves as a proxy indicator of depression severity. The 62 participants were followed for a total of 6330 person-days, with data collection ranging from 5 to 180 days per person. After applying a minimum threshold of one-quarter valid hours of data per day, a total of 5177 person-days was included in the analysis.

**Table 1.** Demographic and clinical characteristics of the participants.

Variables	HC^a^ group (n=5)	BP^b^ group (n=20)	MDD^c^ group (n=27)	*P* value
Demographic characteristics				
Sex, n (%)				.70
Female	11 (73.3)	12 (60)	17 (63)	
Male	4 (26.7)	8 (40)	10 (37)	
Occupation, n (%)				.10
Unemployed	0 (0)	7 (30)	6 (22.2)	
Employed	10 (66.7)	10 (50)	16 (59.3)	
Student	3 (20)	2 (10)	2 (7.4)	
Retired	2 (13.3)	1 (5)	3 (11.1)	
Age, mean (SD)	40.33 (14.57)	40.35 (11.15)	43.19 (11.82)	.68
Clinical characteristics, mean (SD)				
YMRS^d^	—^e^	2.90 (4.34)	0.52 (1.19)	.02
HAM-D^f^	—	7.45 (4.57)	7.16 (5.21)	.81
BDI^g^	4.93 (5.48)	20.84 (10.62)	18.32 (14.37)	<.001
PHQ-4^h^	0.87 (1.18)	5.00 (2.32)	4.52 (3.30)	<.001
BAI^i^	1.60 (2.35)	13.35 (7.97)	13.67 (9.59)	<.001
Follow-up days	126.13 (79.24)	98.00 (83.04)	107.96 (72.96)	.45

a HC: healthy control.

b BP: bipolar disorder.

c MDD: major depressive disorder.

d YMRS: Young Mania Rating Scale.

e Not available.

f HAM-D: Hamilton Depression Rating Scale.

g BDI-II: Beck Depression Inventory.

h PHQ-4: Patient Health Questionnaire-4.

i BAI: Beck Anxiety Inventory.

### Correlation of GPS Indicators and EMA Depressed Mood on Weekdays and Weekends

Table 2 illustrates whether differences in mobility patterns between weekdays and weekends are associated with EMA-measured mood. Figure S4 in Multimedia Appendix 1 shows that significant correlations were found between 6 GPS features and mood performance across various time frames, with daily data demonstrating stronger correlations (*P*<.05) compared to weekly or semi-annual data. These findings highlight the value of daily GPS data in reflecting clinical mood variations. Building on this, subsequent analyses focused on day-by-day GPS data.

To identify the most relevant GPS indicators, we evaluated their effectiveness in determining depressive status on weekdays and weekends. The study included 5177 days of data: 3716 weekdays and 1461 weekends. Multicollinearity was addressed by removing GPS features with a variance inflation factor (VIF) >3. Logistic regression, adjusted for age, sex, and employment status, revealed that on weekdays, lower LV (OR 0.975, 95% CI 0.957‐0.993; *P*=.008) and TT (OR 0.048, 95% CI 0.012‐0.200; *P*<.001) scores were significantly associated with depressive status. On weekends, lower entropy scores (OR 0.662, 95% CI 0.520‐0.842; *P*=.001) were similarly linked to depressive status.

**Table 2.** Logistic regression for distinguishing participants with and without depressive status using GPS features presented in number of person-day.

	Total			Weekdays			Weekends		
	β coefficient	*P* value	OR^a^ (95% CI)	β coefficient	*P* value	OR (95% CI)	β coefficient	*P* value	OR (95% CI)
Sex	−0.636	<.001^b^	0.529 (0.460-0.609)	−0.667	<.001^b^	0.513 (0.435-0.606)	−0.542	<.001^b^	0.582 (0.444-0.762)
Age	−0.019	<.001^b^	0.981 (0.977-0.986)	−0.017	<.001^b^	0.983 (0.977-0.989)	−0.021	<.001^b^	0.979 (0.970-0.988)
Employee or student	−0.360	<.001^b^	0.698 (0.609-0.800)	−0.291	<.001^b^	0.747 (0.635-0.879)	−0.518	<.001^b^	0.595 (0.461-0.770)
Location variance	−0.020	.01^b^	0.980 (0.965-0.995)	−0.026	.008^b^	0.975 (0.957-0.993)	−0.007	.61	0.993 (0.966-1.020)
Speed mean	0.001	.27	1.001 (1.000-1.002)	0.001	.24	1.001 (1.000-1.002)	<0.001	.89	1.000 (0.998-1.003)
Transition time	−2.474	<.001^b^	0.084 (0.028-0.255)	−3.030	<.001^b^	0.048 (0.012-0.200)	−1.454	.09	0.234 (0.043-1.270)
Number of clusters	−0.002	.37	0.998 (0.995-1.002)	−0.001	.51	0.999 (0.994-1.003)	−0.002	.53	0.998 (0.992-1.004)
Homestay	−0.061	.59	0.941 (0.754-1.174)	−0.019	.89	0.981 (0.753-1.278)	−0.117	.58	0.890 (0.590-1.342)
Entropy	−0.191	.004^b^	0.826 (0.725-0.940)	−0.099	.22	0.906 (0.774-1.059)	−0.413	.001^b^	0.662 (0.520-0.842)

a OR: odds ratio.

b Indicates significance at *P* value <.05.

### Difference in Fourier Transform Data Among Patients

**Figure 2.**
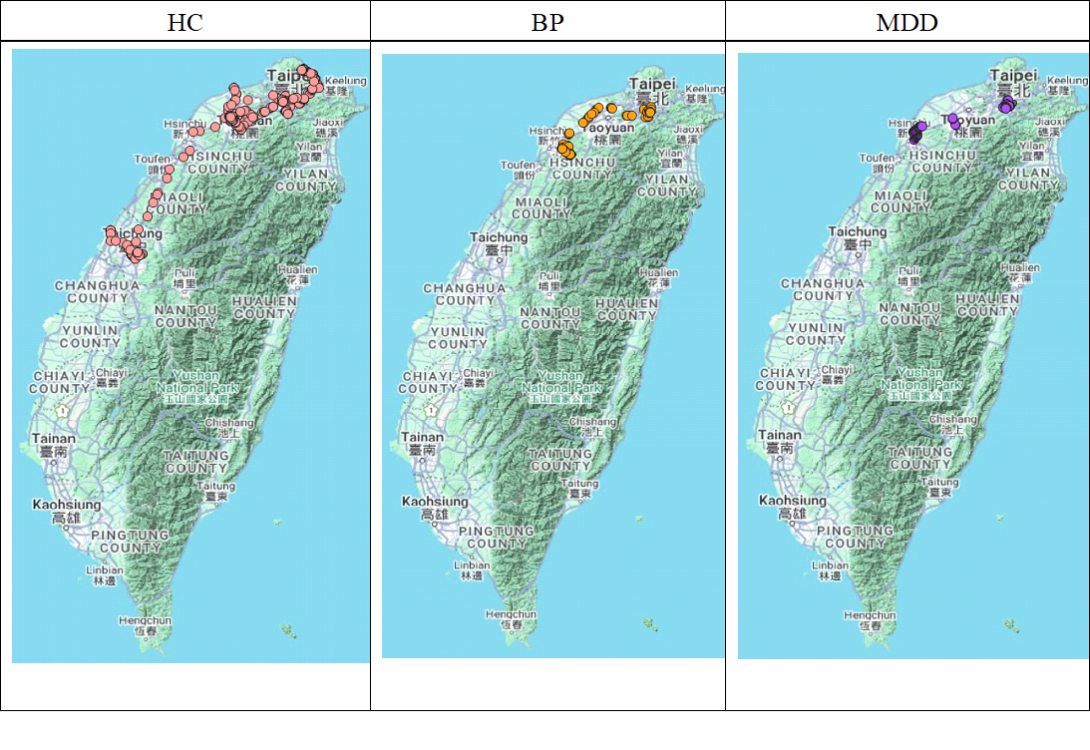
Location distribution map of examples of healthy control (HC), bipolar disorder (BP), and major depressive disorder (MDD) in May 2020.

At the same period within a given month, GPS location data did not distinguish between MDD and BP groups (see Figure 2). To address this, we employed frequency-domain analysis to explore potential diagnostic differences. This analysis focused on LV and entropy, as TT primarily reflects time-domain characteristics. Unlike previous analyses that differentiated GPS indicators by weekdays and weekends, Fourier transform analysis used weekly data for initial exploration.

**Figure 3.**
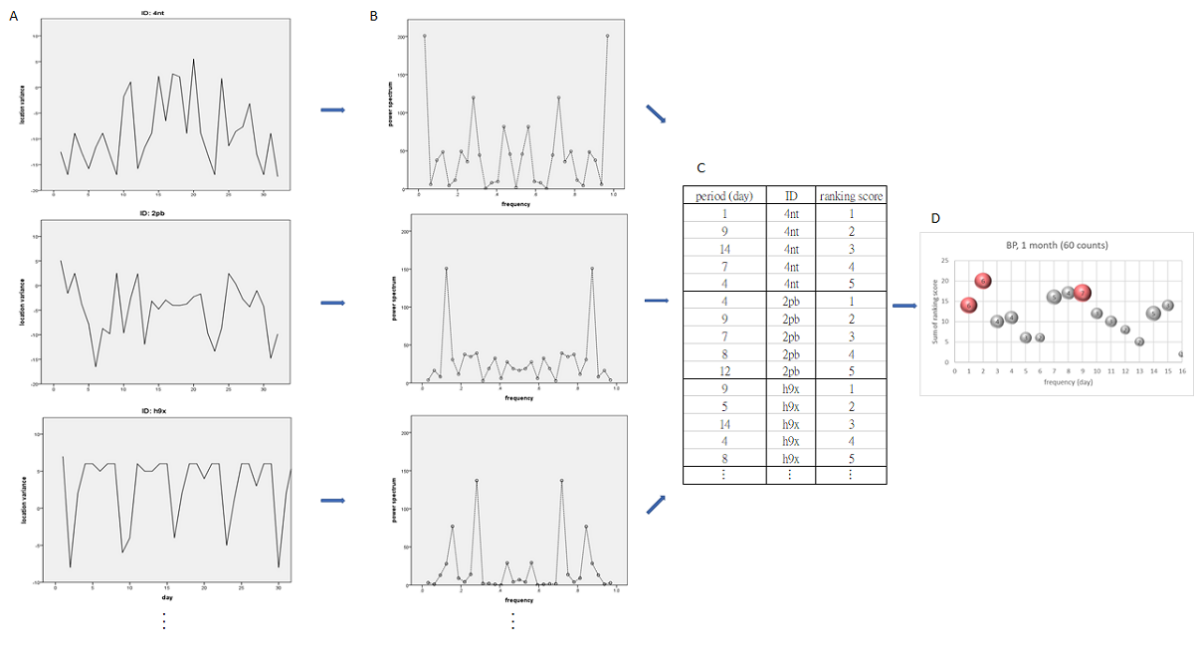
Example of forming the Fourier transform procedure for the bipolar (BP) disorder group.

First, time-domain graphs (see Figure 3A) were plotted for LV and entropy counts in each group. Fourier transforms were then applied to derive frequencies and corresponding power spectra, as shown in frequency-domain graphs (see Figure 3B). Distinct frequency and power spectrum values emerged among groups (see Figure 3C). Periodic waves corresponding to the top five power values were identified, with their summed ranks on the y-axis used to indicate intensity (see Figure 3D). Lower ranks reflected stronger intensity, highlighting shared periodic waves (x-axis) among participants.

The period indicates the reciprocal of the frequency. The ranking score indicates the power spectrum in descending order from the maximum value.

**Figure 4.**
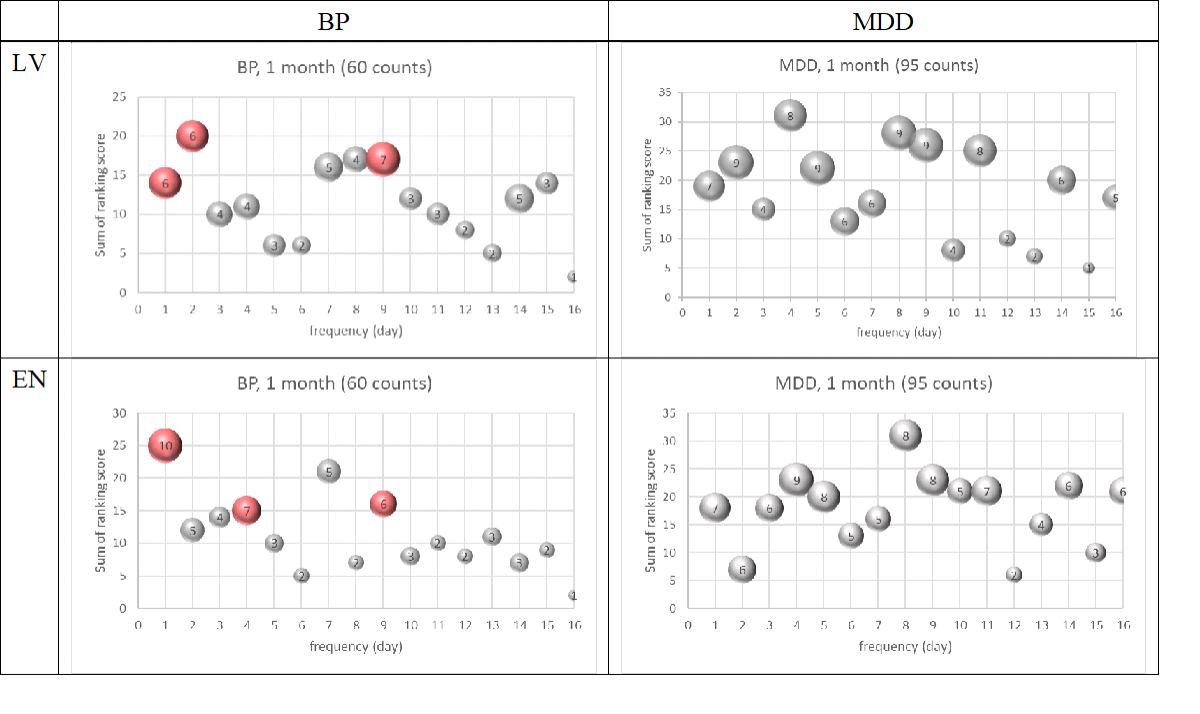
Bubble plot of top 5 location variance (LV) and entropy (EN) Fourier power spectrum ranking score in number of person-frequency. BP: bipolar disorder; MDD: major depressive disorder.

Figure 4 shows differences in periodic waves between groups for LV and entropy. Among participants with BP, more than half showed periodic waves at 1-day, 2-day, and 9-day cycles, whereas no clear periodic cycles were observed for MDD. Similarly, for entropy, participants with BP exhibited cyclic waves at 1-day, 4-day, and 9-day cycles on a monthly basis, while participants with MDD lacked consistent periodicity. Results for quarterly and bi-monthly periods are detailed in Figures S5 and S6 in Multimedia Appendix 1.

In Figure 4, the numbers in the circle indicate the number of people with this frequency. Colored circles indicate more than 50% of participants have this frequency.

Amplitude-based power spectrum analysis further revealed significant differences in maximum power spectra for LV (*P*=.04) and entropy (*P*=.02) between BP and MDD groups (see Table 3). Intensity rankings for entropy demonstrated its superior efficacy over LV in distinguishing diagnoses. These results suggest that while BP and MDD share similar periodic cycles, the intensity and stability of mobility patterns are more diagnostic. We found no significant differences in power spectra and frequencies between HC and BP, and between HC and MDD (see Tables S2 and S3 in Multimedia Appendix 1).

**Table 3.** Mann-Whitney *U* test for distribution of Fourier frequency and power spectrum of location variance and entropy between bipolar disorder (BP) and major depressive disorder (MDD) groups in 1 month.

	Location variance			Entropy		
	BP^a^ (n=12)	MDD^b^ (n=19)	*P* value	BP (n=12)	MDD (n=19)	*P* value
Power spectrum						
Maximum	20.17	13.37	.04**^c^**	20.71	13.03	.02^c^
Second	19.25	13.95	.12	19.96	13.50	.05^c^
Third	19.75	13.63	.07	20.12	13.39	.04^c^
Fourth	19.33	13.89	.11	20.21	13.34	.04^c^
Fifth	19.08	14.05	.14	17.29	15.18	.54
Frequency						
Maximum	15.54	16.29	.83	17.79	14.87	.39
Second	13.67	17.47	.27	13.12	17.82	.16
Third	19.42	13.84	.10	11.58	18.79	.03
Fourth	15.42	16.37	.80	12.54	18.18	.09
Fifth	14.67	16.84	.54	15.38	16.39	.77

a BP: bipolar disorder.

b MDD: major depressive disorder.

c Indicates *P* values <.05.

To evaluate affective mood status, logistic regression was performed on the power spectra of LV and entropy over a month in BP and MDD groups (see Table 4). Exposure was the power of LV or entropy, and the outcome was with or without EMA mood. Without covariate adjustments, depressive status (OR 0.998, 95% CI 0.995‐0.999; *P*=.02), manic status (OR 0.992, 95% CI 0.986‐0.999; *P*=.03), and irritability (OR 0.996, 95% CI 0.992‐0.999; *P*=.02) were inversely associated with LV power. Manic (OR 0.249, 95% CI 0.079‐0.781; *P*=.02) and irritability (OR 0.364, 95% CI 0.162‐0.819; *P*=.02) statuses correlated with lower entropy power. After adjusting for age, gender, and employment status, significant differences were observed only in depressive status for LV power (OR 0.9976, 95% CI 0.9956‐0.9996; *P*=.02). After adjusting for age, gender, employment status, and antidepressant use, LV power remained significantly associated with depressive status (OR 0.998, 95% CI 0.995‐0.999; *P*=.02). This indicates that decreased movement intensity is a key marker of depressive status, with LV power outperforming entropy in differentiating mood states. Overall, the power spectrum of cyclic waves provides a valuable metric for representing human mobility patterns and differentiating mood disorders.

**Table 4.** Distinguishing the affective state of participants with BP and MDD using Fourier power spectrum of location variance (LV) and entropy.

	Model 1^a^			Model 2^b^			Model 3^c^			Model 4^d^		
	β coefficient	*P* value	OR^e^ (95% CI)	β coefficient	*P* value	OR (95% CI)	β coefficient	*P* value	OR (95% CI)	β coefficient	*P* value	OR (95% CI)
Fourier spectrum power of LV												
With or without fatigue	0.001	.11	1.001 (1.000-1.003)	0.001	.12	1.001 (1.000-1.003)	0.002	.10	1.002 (1.000-1.004)	0.002	.13	1.002 (1.000-1.004)
With or without depression	−0.002	.02^f^	0.998 (0.996-1.000)	−0.003	.01^f^	0.997 (0.996-0.999)	−0.002	.02^f^	0.998 (0.996-1.000)	−0.002	.02^f^	0.998 (0.996-1.000)
With or without mania	−0.008	.03^f^	0.992 (0.986-0.999)	−0.003	.42	0.997 (0.989-1.005)	−0.004	.33	0.996 (0.988-1.004)	−0.005	.23	0.995 (0.986-1.003)
With or without irritability	−0.004	.02^f^	0.996 (0.992-0.999)	−0.001	.32	0.999 (0.996-1.001)	−0.001	.43	0.999 (0.996-1.002)	−0.001	.57	0.999 (0.996-1.002)
Fourier spectrum power of entropy												
With or without fatigue	−0.210	.45	0.811 (0.472-1.392)	0.012	.97	1.012 (0.567-1.807)	0.026	.93	1.026 (0.574-1.833)	0.285	.38	1.330 (0.704-2.512)
With or without depression	−0.306	.26	0.736 (0.431-1.259)	−0.156	.58	0.855 (0.494-1.481)	−0.121	.66	0.886 (0.512-1.532)	−0.064	.82	0.938 (0.541-1.626)
With or without mania	−1.390	.02^f^	0.249 (0.079-0.781)	−1.123	.08	0.325 (0.092-1.147)	−1.071	.10	0.343 (0.095-1.234)	−0.696	.30	0.499 (0.133-1.872)
With or without irritability	−1.009	.02^f^	0.364 (0.162-0.819)	−0.775	.09	0.461 (0.189-1.122)	−0.683	.14	0.505 (0.206-1.241)	−0.218	.66	0.804 (0.307-2.109)

a Model 1: no adjustment.

b Model 2: adjusted for age and sex.

c Model 3: adjusted for age, sex, and work.

d Model 4: adjusted for age, sex, work, and antidepressants. Fatigue, depression, mania, and irritability status was defined as EMA score >1. EMA score=1 means without the affective status.

e OR: odds ratio.

f *P*<.05.

## Discussion

### Principal Findings

By leveraging frequency-domain analysis, it provides insights into the periodicity and intensity of mobility behaviors in mood disorders. Patients with BP exhibit stronger periodic mobility patterns than patients with MDD. In addition, entropy frequency data over a 1-month period effectively distinguished BP from MDD diagnoses, while the Fourier spectrum power of LV distinguished affective status. Mobility indicators, including LV, TT, and entropy, showed significant associations with depressed mood states. Lower LV and TT values were observed on weekdays, while reduced entropy occurred on weekends (see Table 2). Clinically, these patterns suggest that depression is associated with reduced mobility (lower LV and TT) and less variability in daily routines (diminished entropy), potentially reflecting decreased motivation and social engagement. Notably, the frequency-domain features of entropy and LV may hold diagnostic relevance: first, entropy frequency patterns could reflect mood instability specific to BP, distinguishing it from MDD. Second, LV spectral power might serve as a real-time indicator of current mood state severity. These digital behavioral signatures highlight the potential for passive monitoring tools to objectively track mood disorders and inform personalized interventions.

This study investigated the correlation between GPS mobility patterns and clinical symptoms, EMA-reported mood states, and diagnostic differences in patients with MDD and BP using Fourier transform analysis. Our findings align with previous studies, confirming that GPS mobility data can effectively detect depressive symptoms [12,35]. Movement data play a critical role in inferring mental health symptoms, with reduced mobility being associated with lower severity of depression [5]. Mobility metrics such as LV, entropy, and TT showed negative correlations with BDI, PHQ-4, and HAMD scores, consistent with earlier research [36]. In addition, reduced GPS mobility, including fewer clusters, correlated with higher PHQ-4 scores, reflecting diminished motivation [10]. Similarly, entropy, TT, and NC were negatively associated with BAI scores, suggesting that participants with higher anxiety scores exhibited less regular daily activities, reduced movement, and increased social withdrawal [8].

Our analysis showed that daily GPS data better captured correlations with clinical mood data than weekly or semi-annual aggregates. Aggregating over extended periods can obscure meaningful variations, such as those caused by seasonal changes or holidays, diluting the significance of extreme movements. Daily analyses allow for a clearer understanding of individual routines and mobility patterns.

This study also evaluated the effectiveness of common GPS features on weekdays and weekends in relation to EMA-reported depressive mood. Specifically, for monitoring short-term daily emotional fluctuations, indicators such as LV, TT, and entropy demonstrated heightened sensitivity, capturing even subtle changes. The relationship between these GPS features and EMA depressive mood varied between weekdays and weekends. On weekdays, smaller LV and TT values were significantly associated with higher EMA depressive scores, while on weekends, lower entropy values were linked to increased depressive mood. Features related to location, such as LV and entropy, consistently showed strong associations with depressive symptoms, corroborating findings from previous research [2]. Participants with higher EMA depressive scores displayed fewer positional changes, lower overall GPS activity, and decreased entropy, indicating greater variability in location use [6]. Typically, diagnostic methods fail to capture real-time mood fluctuations or stability, as emotional changes often stem from shifts in preceding and subsequent EMA assessments. Interestingly, while previous studies [19] emphasized the importance of entropy and LV on weekends, our findings suggest that LV and TT are more relevant on weekdays. This distinction could be attributed to weekday mobility patterns being influenced by social roles, making LV and TT more reliable indicators during these periods. On the other hand, entropy, which reflects the diversity of time spent in location clusters, was more significant on weekends, likely due to the role of interpersonal relationships and social interactions shaping weekend mobility behaviors.

This study also introduced an innovative application of frequency domain analysis to differentiate GPS variations. Fourier-transformed data for LV and entropy revealed 2 key indicators: frequency magnitude and power spectrum size. While periodic waves alone did not effectively distinguish diagnoses, the power spectrum can serve as a reliable marker to distinguish BP from MDD. Patients with BP exhibited greater power spectra intensity and robustness in both LV and entropy compared to patients with MDD, reflecting more complex activity patterns. LV and entropy can represent both spatial variability and the temporal distribution at the same location, making them effective measures for distinguishing diagnoses. Previous studies support these findings, indicating that activity metrics such as entropy and regularity indices in patients with BP provide deeper insights into activity patterns. As digital activity markers, these metrics aid in distinguishing patients with BP from patients with MDD [11]. Consistently, entropy emerged as a diagnostic marker, with depressed groups spending more time in single location clusters and visiting fewer varied locations [37]. Using 3 different time spans for Fourier transformations—1 month, 2 months, and a season—our study revealed that a 1-month tracking period provided the strongest correlations, highlighting the efficacy of this time span for frequency-domain analysis. Collecting LV and entropy data over 1 month effectively distinguished emotional symptoms and diagnoses without requiring longer durations, such as a season or half-year. Furthermore, the absence of significant cyclic waves in distinguishing BP from MDD suggests that both clinical groups may benefit from monthly clinical visits as an optimal threshold. Short-time Fourier transform parameters proved to be the most robust and least noise-sensitive features for frequency-domain analysis [38]. These findings underscore the potential of Fourier transformations as essential tools for future diagnostics, emotional state assessments, and treatment planning.

GPS data provide an objective measure of patients’ daily mobility patterns, reducing recall bias compared to traditional self-reports like questionnaires and interviews. This study is methodologically innovative, leveraging Fourier-transformed GPS mobility data to analyze temporal dynamics of movement patterns in mood disorders. We found that patients with MDD tend to exhibit a lower mobility power spectrum, while patients with BP show a broader power spectrum and more frequent movement patterns. Therefore, GPS data can serve as an early indicator of relapse risk, enabling clinical intervention when the system detects potential signs of recurrence.

This study has several limitations. First, observations from the same individual on different days resulted in unequal contributions across participants, leading to data weight bias. For example, the person-day analysis in Table 2 did not account for repeated measurements, and logistic regression assumed independence between different days for the same participant. Previous study indicates that within-subject variability (day-to-day) constitutes a larger proportion of total variability than between-subject variability [39]. Although the large number of days used across participants maintained consistent results, the relatively small sample size made repeated measurements riskier, as the number of repeated days exceeded the number of participants. Second, there is a potential selection bias in the recruitment of both patients and healthy controls. Healthy controls recruited near a hospital may not represent the general population, as they could have distinct socioeconomic backgrounds, greater health awareness, or more frequent medical visits. This may introduce selection bias, as such individuals might have a higher prevalence of chronic conditions or health care–seeking behaviors. Consequently, findings may not generalize to mood disorder patients outside hospital settings or a truly random sample of healthy individuals. In addition, patients with MDD and BP were recruited not during the acute phase, and the majority (42/47, 89.4%) were receiving ongoing treatment, which may have resulted in fewer mood fluctuations, limiting the generalizability of the findings. After months of repeated prompts, participants may develop survey fatigue, leading to less accurate or habitual responses (eg, defaulting to neutral ratings). While EMA minimizes recall bias by design, prolonged studies risk fatigue-induced lapses, where participants resort to heuristic judgments (eg, averaging moods over hours) rather than real-time reporting. If participants with severe mood instability stop responding, the final sample may overrepresent stable individuals, biasing conclusions about mood trajectories. Compliance rates often drop in longitudinal studies due to participant burnout—non-random drop-out introduces attrition bias. Third, movement estimates from smartphones can be influenced by several factors, including device status (eg, turned off, low battery, and turning on airplane mode), GPS or network usage habits, and international travel. These factors could introduce estimation biases. Fourth, GPS data collected from iPhones in this study was more extensive and covered broader ranges than data from Android devices. However, the type of phone may correlate with variables such as socioeconomic background, introducing potential confounding effects. Fifth, while wearable devices, such as the Apple Watch, are used for applications like heart rate monitoring in elderly populations, their use in psychiatry remains limited. Incorporating wearable devices in future research could provide complementary data and enhance the robustness of mobility-based analyses. Finally, residual confounding effects from differences in psychopharmacological treatment regimens (eg, mood stabilizers and antipsychotics) between patients with BP and MDD cannot be fully excluded, despite adjustment for antidepressant use in our models.

### Conclusion

By integrating frequency-domain analysis with traditional mobility metrics, this study enhances the precision of digital phenotyping, paving the way for more objective, data-driven assessments of mood disorders. Our study results highlight the potential of GPS mobility data as a valuable tool for diagnosing and monitoring mood disorders, particularly in distinguishing BP from MDD and assessing emotional fluctuations via EMA. On weekdays, LV and TT were effective indicators of depressive states, while entropy proved more relevant on weekends. Fourier transform analysis revealed that entropy frequency data over a 1-month span was especially effective in differentiating BP from MDD, with patients with BP exhibiting higher power spectrum intensity and complexity in mobility patterns than patients with MDD. LV provided additional insights into affective status. Compared to cyclical waves, the amplitude of LV and entropy frequencies better captured movement intensity, offering a more detailed understanding of mobility patterns. Daily GPS data proved superior in capturing mood-related variations, emphasizing the importance of selecting appropriate time spans to optimize diagnostic accuracy. Integrating GPS-based digital phenotyping with traditional assessments holds significant promise for advancing real-time mental health monitoring and improving the diagnostic process for BP and MDD.

## Supplementary material

10.2196/71658 Multimedia Appendix 1 Supplementary tables and figures.

## Abbreviations

BAI: Beck Anxiety Inventory
BDI: Beck Depression Inventory
BP: bipolar disorder
EMA: ecological momentary assessment
HAM-D: Hamilton Rating Scale for Depression
HC: healthy control
LV: location variance
MDD: major depressive disorder
NC: number of clusters
PHQ-4: Patient Health Questionnaire-4
SM: speed mean
TT: transition time
