# Supplementary material for: Fourier Transform Analysis of GPS-Derived Mobility Patterns for Diagnosis and Mood Monitoring of Bipolar and Major Depressive Disorders: Prospective Study
Source: J Med Internet Res. 2025 Jul 15;27:e71658. doi: 10.2196/71658 (PMC12287981; doi:10.2196/71658)
Supplement: Multimedia Appendix 1 [file jmir-v27-e71658-s001.docx]

Multimedia Appendix

Figure S1. The screen of the Chinese version of the Beiwe app.


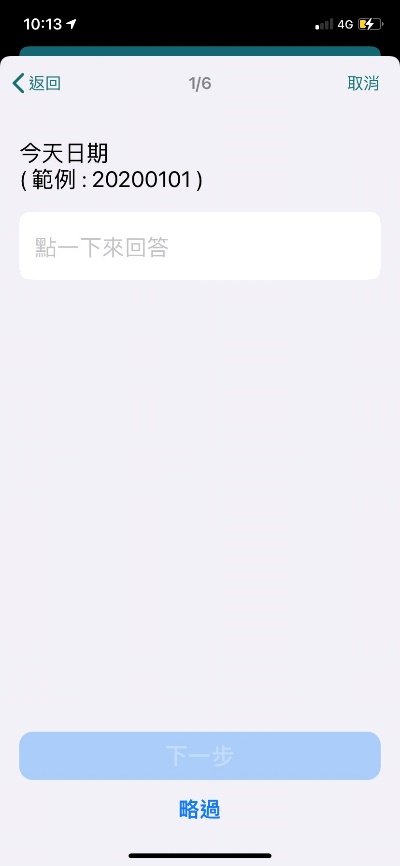

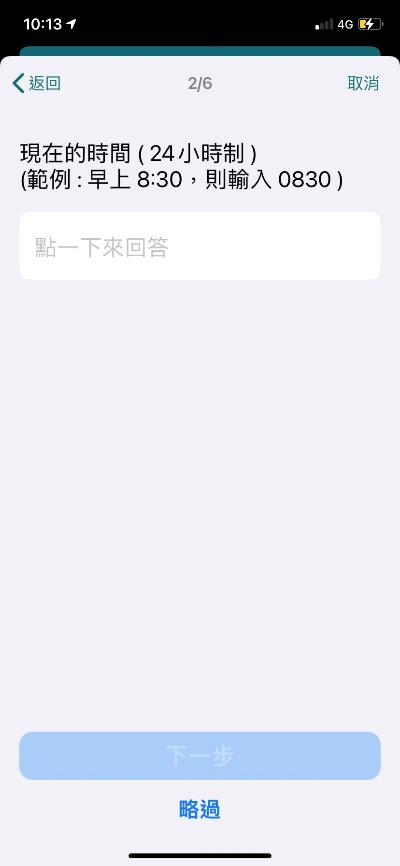

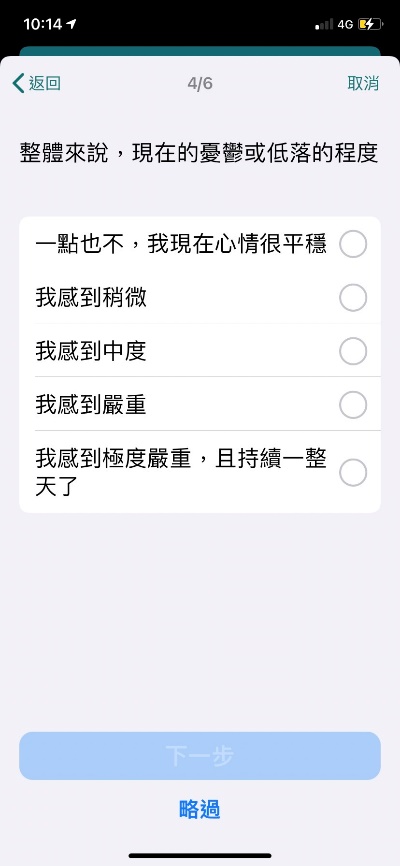


Figure S2. Distribution of GPS raw data from Monday to Sunday during the study period of subject M01.


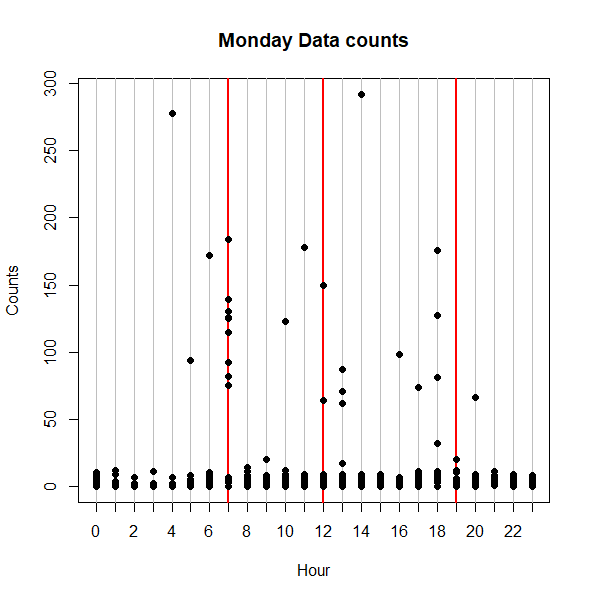

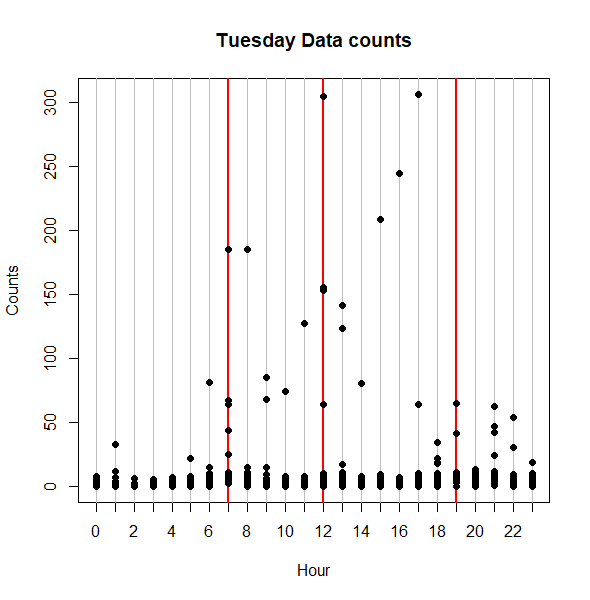

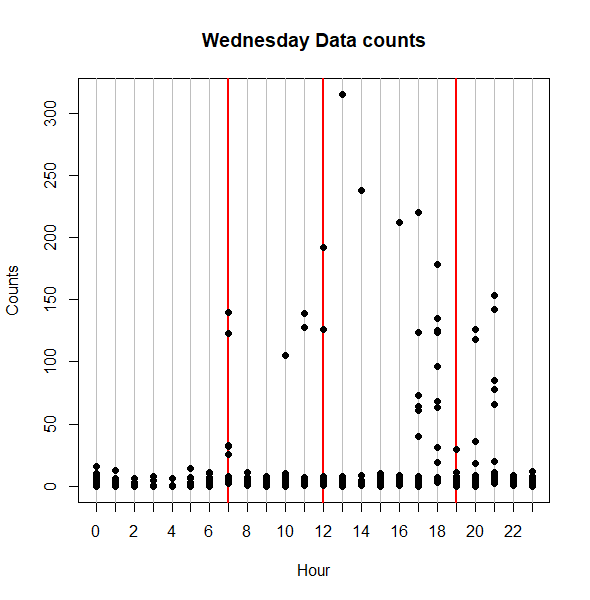

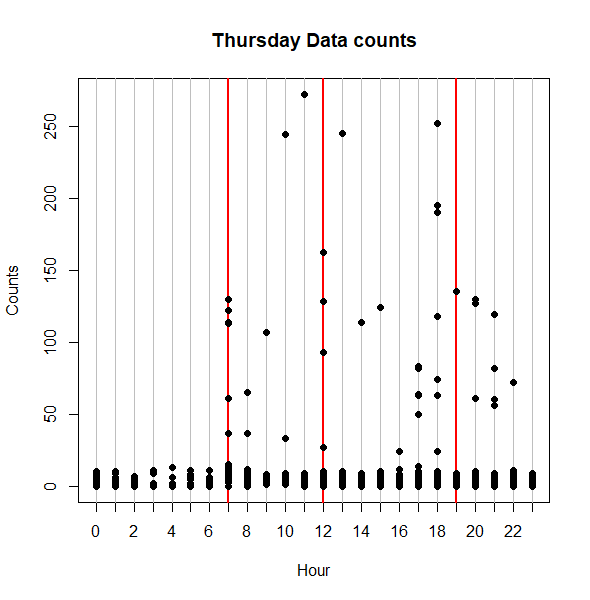

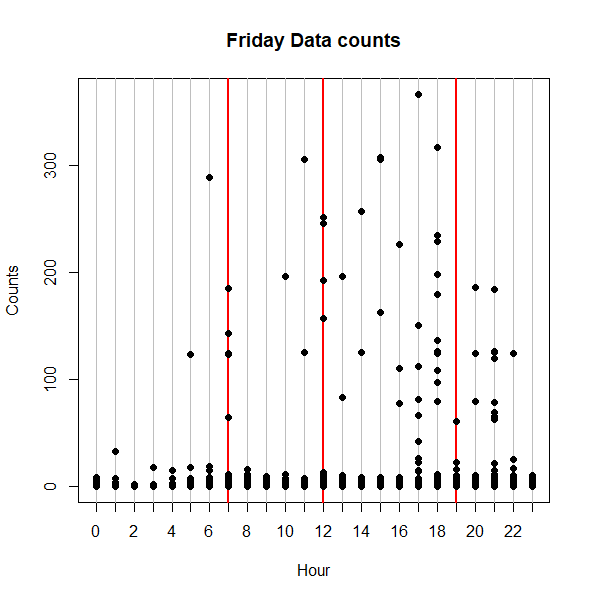

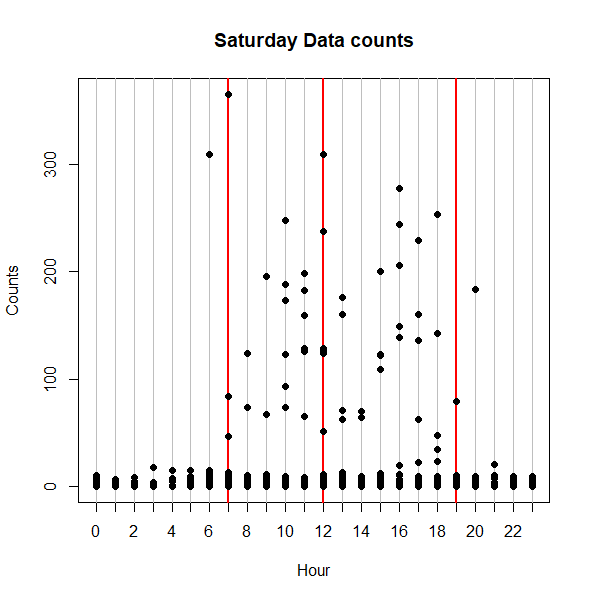

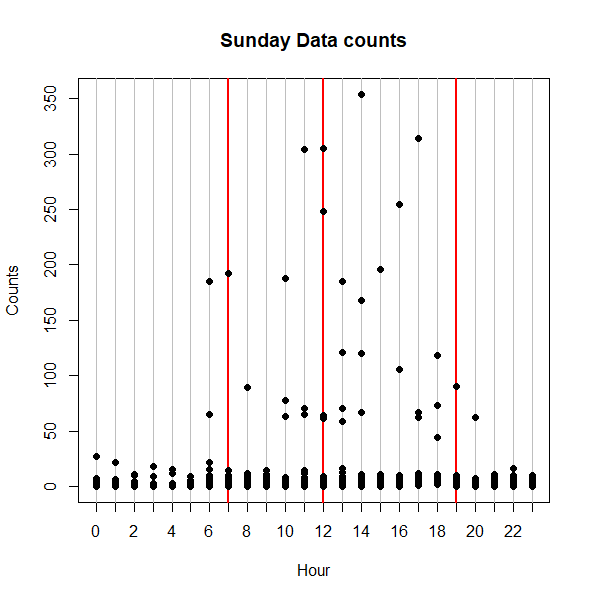


The x-axis represents the 24-hour day, while the y-axis indicates the number of data points recorded for each hour.

Figure S3. GPS tracking of ten simulations in a patient.

| 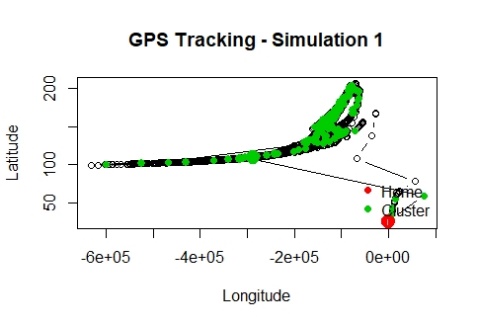 | 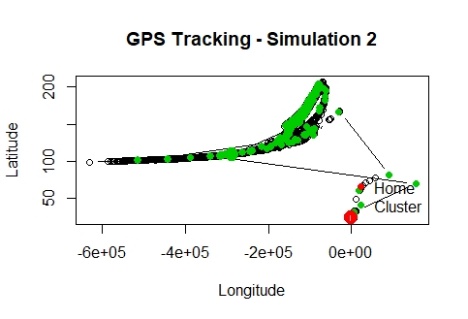 |
| --- | --- |
| 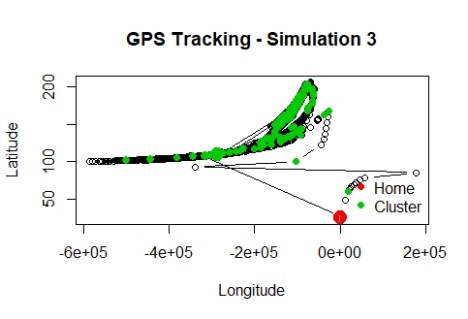 | 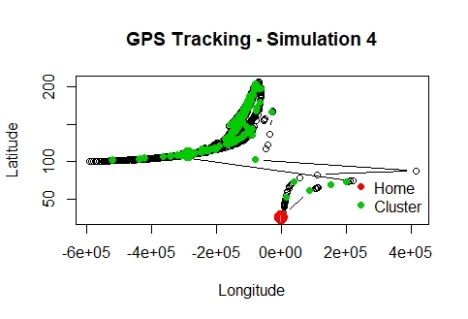 |
| 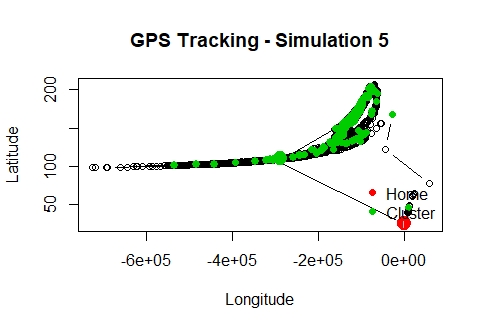 | 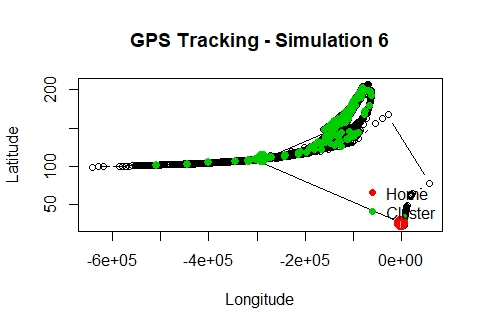 |
| 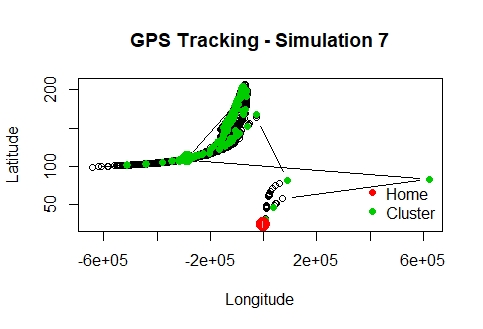 | 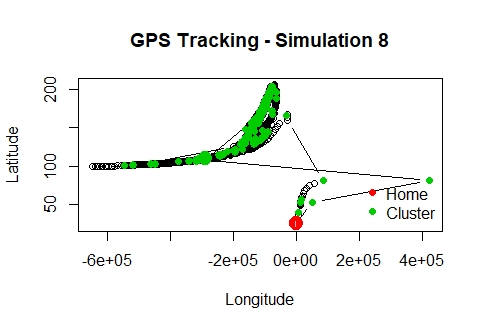 |
| 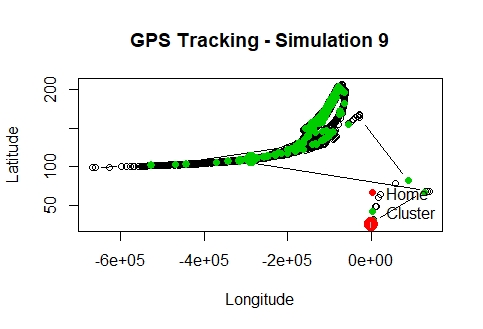 | 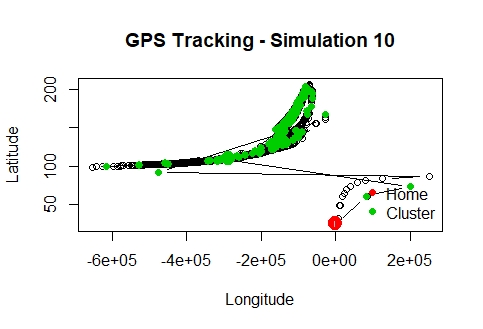 |

Table S1. All EMA items and response.

| items | *(1) How fatigued do you feel right now?* |
| --- | --- |
|  | *(2) How depressed do you feel right now?* |
|  | *(3) How elated do you feel right now?* |
|  | *(4) How irritable/angry do you feel right now?* |
| *Responses* | *1 = Not at all, I’m in a good mood now* |
|  | *2 = A little bit* |
|  | *3 = Moderate* |
|  | *4 = Serious* |
|  | *5 = Serious and lasted all day* |

Figure S4. The relationship between five GPS indicators and clinical scores.

Red indicates a positive correlation, and blue indicates a negative correlation. The solid line represents P value < 0.05. Color shades represent the strength of the correlation.

Figure S5. Bubble plot of top 5 location variance (LV) Fourier power spectrum ranking score in number of person-frequency.

| LV | 4 months | 2 months | 1 month |
| --- | --- | --- | --- |
| HC | 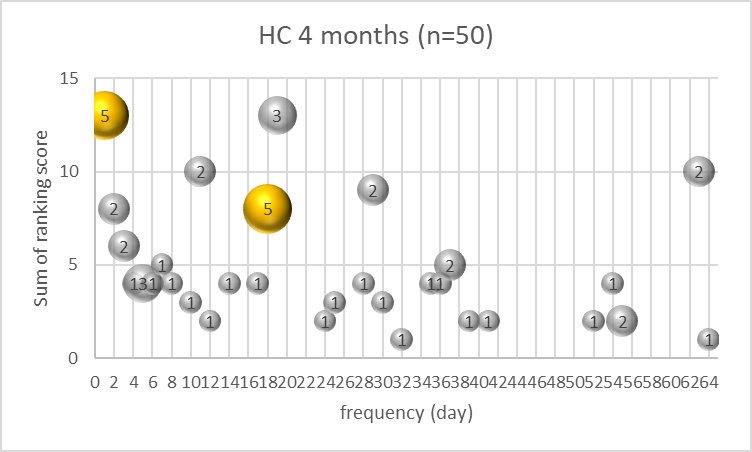 | 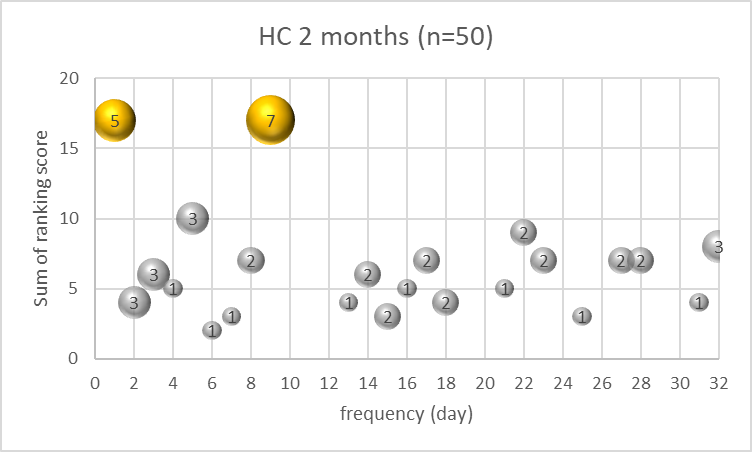 | 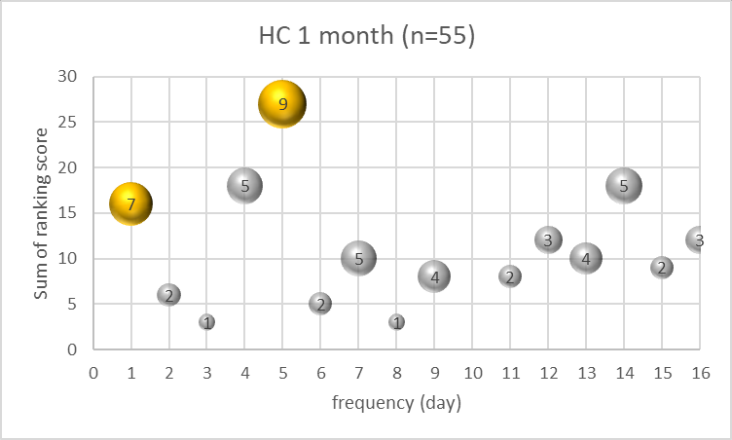 |
| BP | 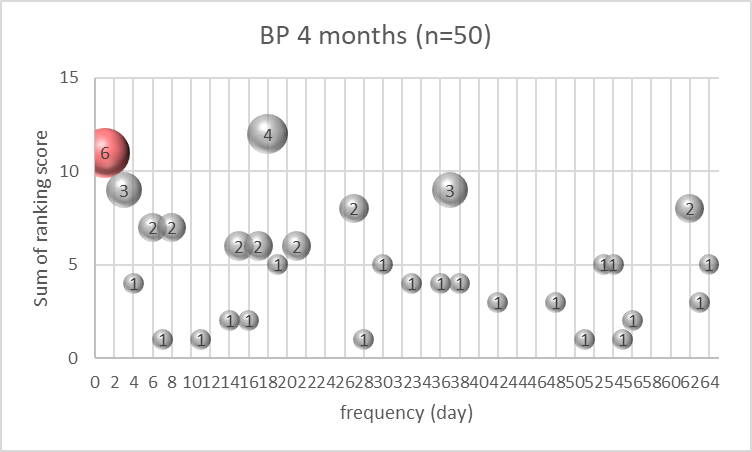 | 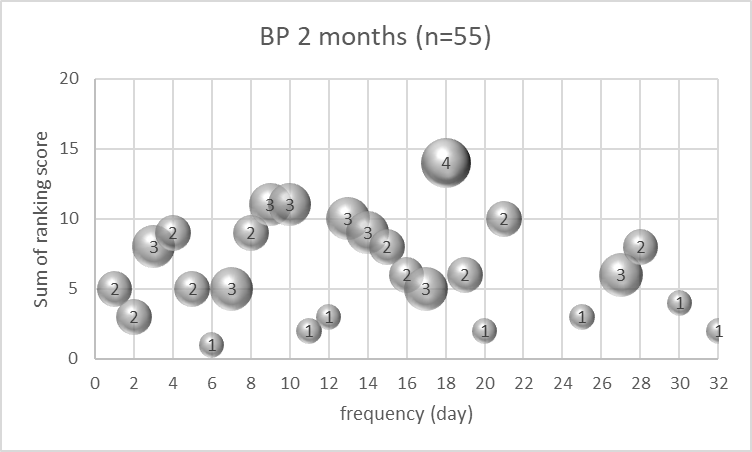 | 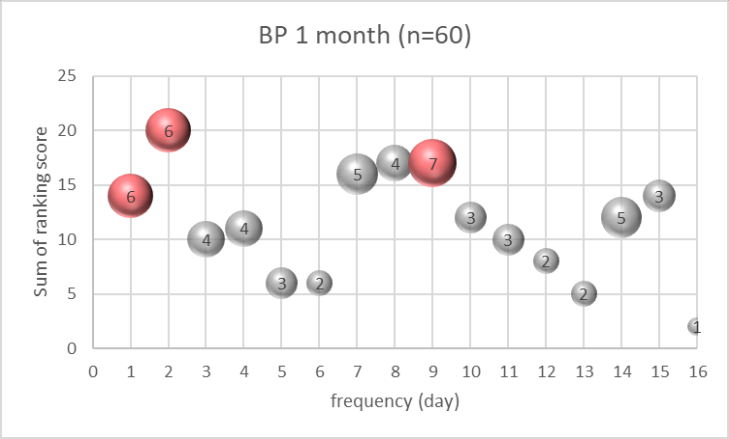 |
| MDD | 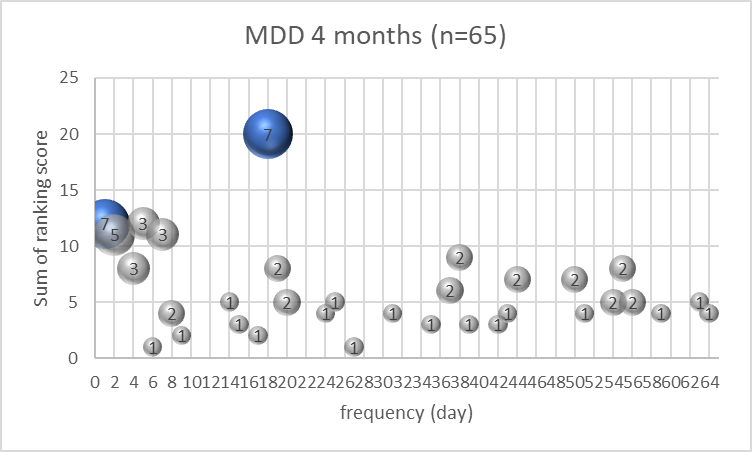 | 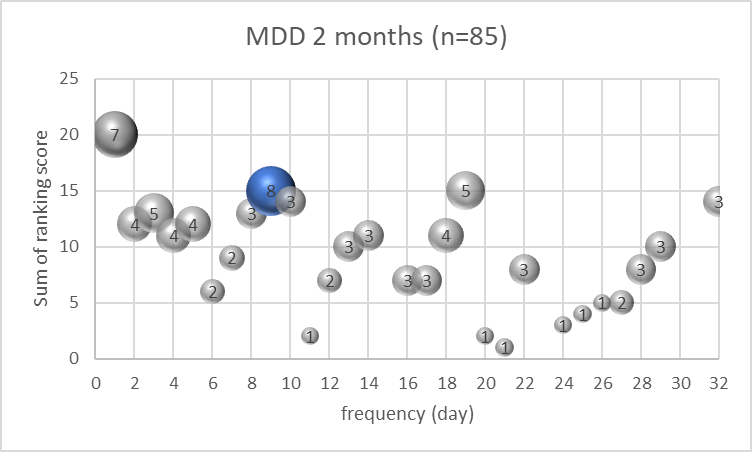 | 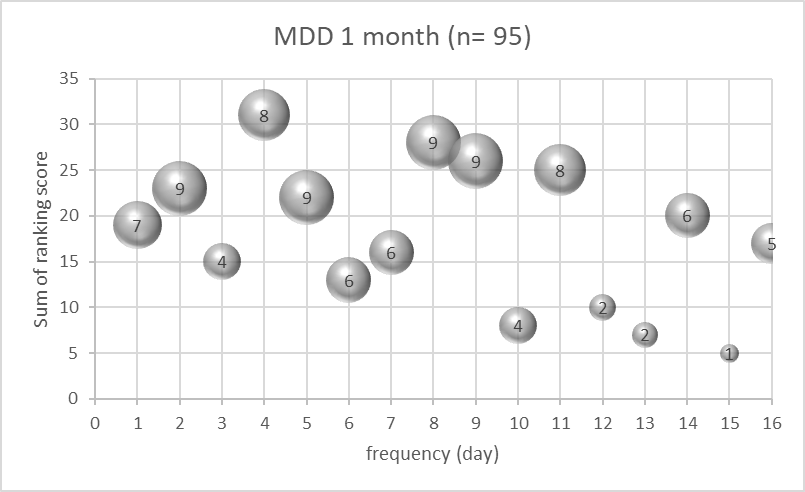 |

Numbers in the circle indicate the number of people with this frequency. Colored Circles indicate more than 50% of participants have this frequency.

Figure S6. Bubble plot of top 5 entropy (EN) Fourier power spectrum ranking score in number of person-frequency.

| EN | 4 months | 2 months | 1 month |
| --- | --- | --- | --- |
| HC | 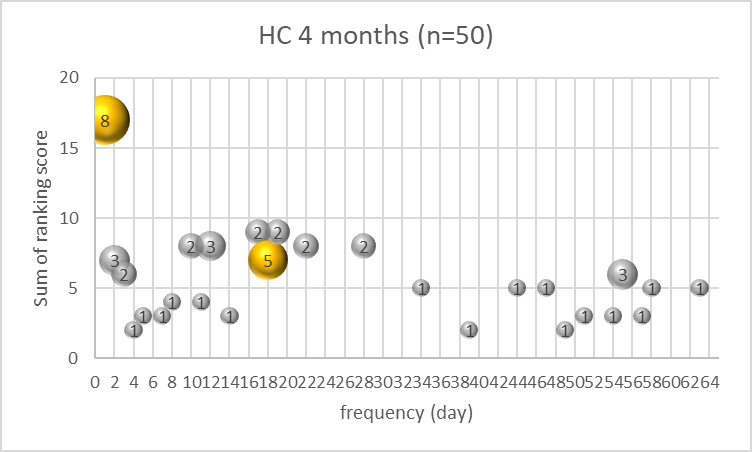 | 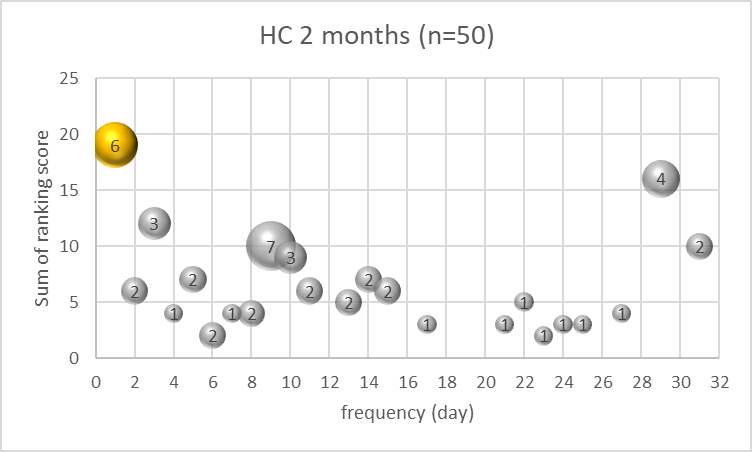 | 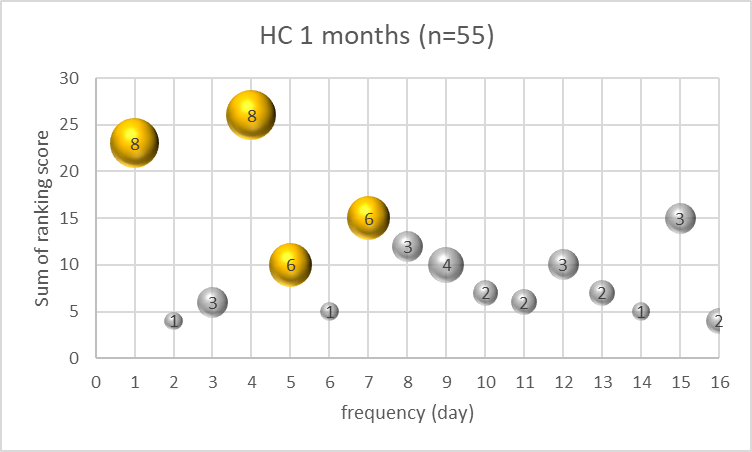 |
| BP | 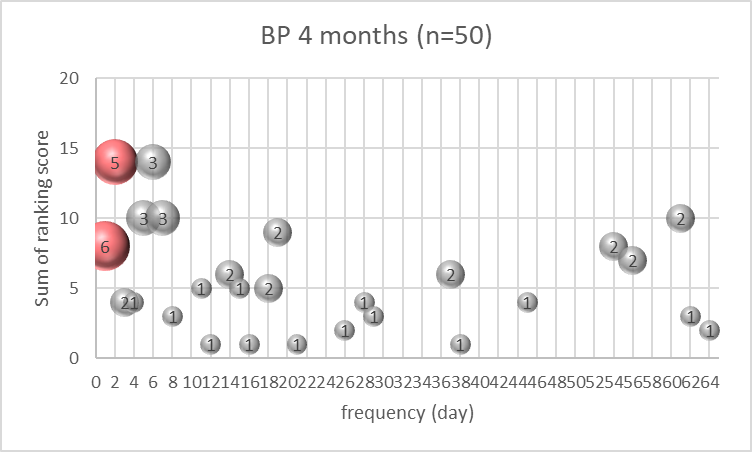 | 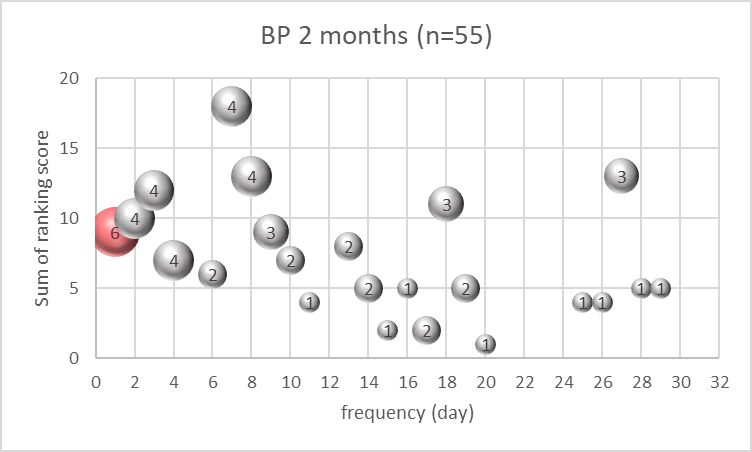 | 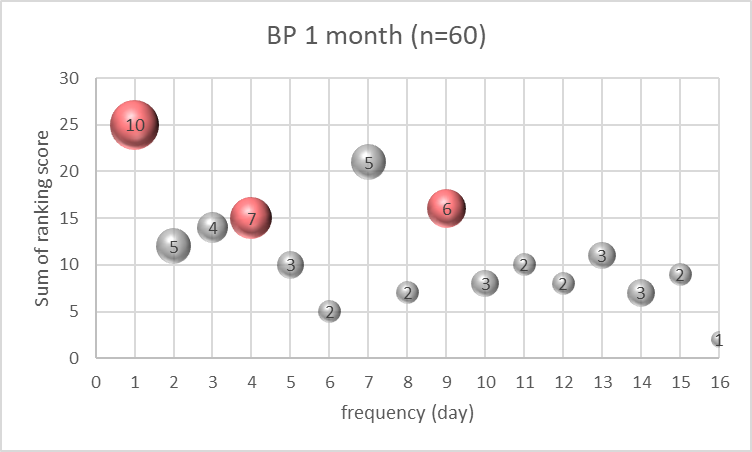 |
| MDD | 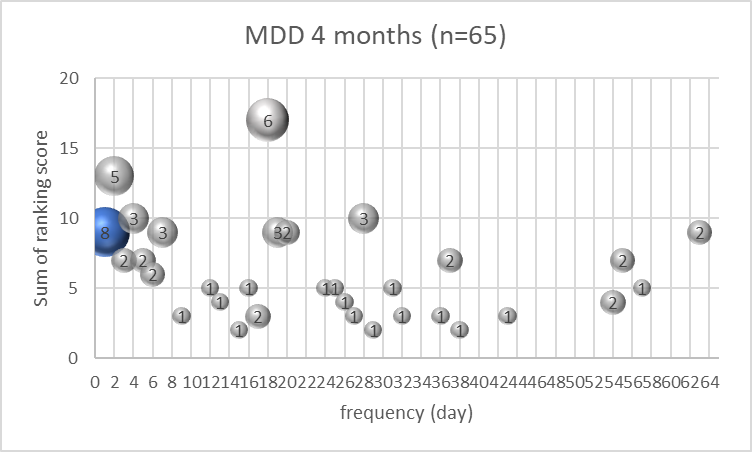 | 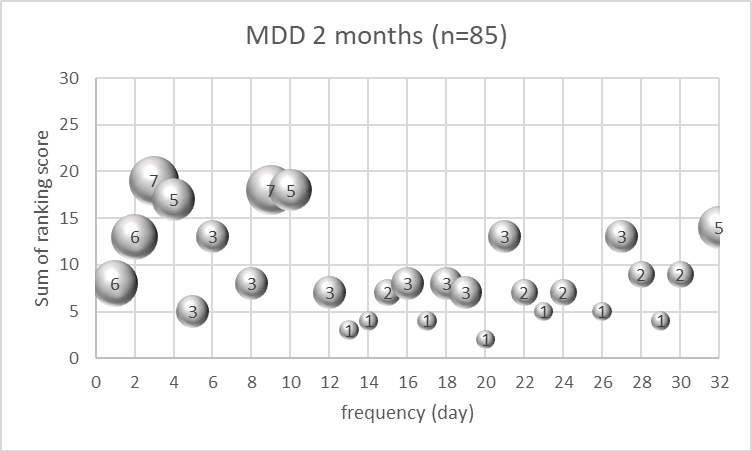 | 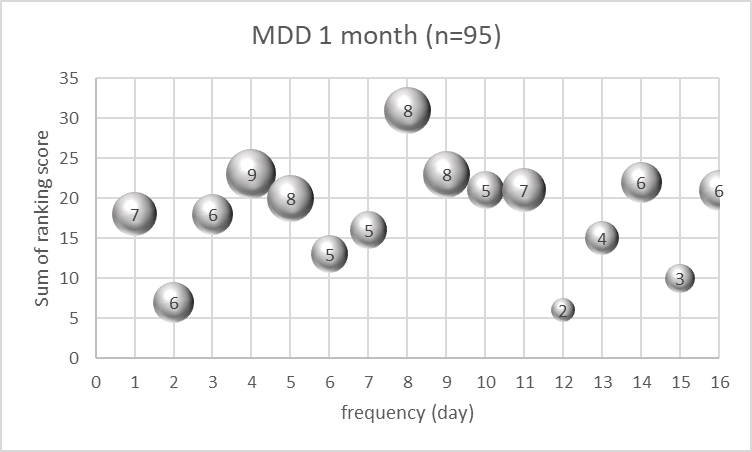 |

Numbers in the circle indicate the number of people with this frequency. Colored Circles indicate more than 50% of participants have this frequenc

Table S2. Mann-Whitney U test for distribution of Fourier frequency and power spectrum of LV/EN between HC and BP groups in 1 month.

|  | Location variance | | |  | Entropy | | |
| --- | --- | --- | --- | --- | --- | --- | --- |
|  | HC (n=11) | BP (n=12) | *P* |  | HC (n=11) | BP (n=12) | *P* |
| Power spectrum |  |  |  |  |  |  |  |
| max | 12.45 | 11.58 | 0.786 |  | 10.36 | 13.50 | 0.288 |
| 2nd | 12.64 | 11.42 | 0.695 |  | 11.18 | 12.75 | 0.608 |
| 3rd | 12.45 | 11.58 | 0.786 |  | 10.55 | 13.33 | 0.347 |
| 4rd | 12.36 | 11.67 | 0.833 |  | 10.18 | 13.67 | 0.235 |
| 5rd | 12.09 | 11.92 | 0.976 |  | 11.00 | 12.92 | 0.525 |
| Frequency |  |  |  |  |  |  |  |
| max | 12.09 | 11.92 | 0.976 |  | 12.05 | 11.96 | 0.976 |
| 2nd | 12.18 | 11.83 | 0.928 |  | 12.64 | 11.42 | 0.695 |
| 3rd | 10.64 | 13.25 | 0.379 |  | 13.77 | 10.38 | 0.235 |
| 4rd | 13.68 | 10.46 | 0.260 |  | 13.86 | 10.29 | 0.211 |
| 5rd | 13.05 | 11.04 | 0.487 |  | 10.73 | 13.17 | 0.413 |

Note: Bold words indicated p-values < 0.05.

Table S3. Mann-Whitney U test for distribution of Fourier frequency and power spectrum of LV/EN between HC and MDD groups in 1 month.

|  | Location variance | | |  | Entropy | | |
| --- | --- | --- | --- | --- | --- | --- | --- |
|  | HC (n=11) | MDD (n=19) | *P* |  | HC (n=11) | MDD (n=19) | *P* |
| Power spectrum |  |  |  |  |  |  |  |
| max | 19.00 | 13.47 | 0.103 |  | 16.91 | 14.68 | 0.525 |
| 2nd | 18.91 | 13.53 | 0.112 |  | 16.27 | 15.05 | 0.735 |
| 3rd | 19.45 | 13.21 | 0.064 |  | 14.73 | 15.95 | 0.735 |
| 4rd | 18.91 | 13.53 | 0.112 |  | 15.45 | 15.53 | 1.000 |
| 5rd | 18.27 | 13.89 | 0.200 |  | 15.18 | 15.68 | 0.899 |
| Frequency |  |  |  |  |  |  |  |
| max | 15.27 | 15.63 | 0.933 |  | 18.27 | 13.89 | 0.200 |
| 2nd | 13.36 | 16.74 | 0.328 |  | 12.95 | 16.97 | 0.232 |
| 3rd | 15.73 | 15.37 | 0.933 |  | 14.18 | 16.26 | 0.553 |
| 4rd | 18.14 | 13.97 | 0.216 |  | 15.86 | 15.29 | 0.866 |
| 5rd | 16.18 | 15.11 | 0.767 |  | 13.18 | 16.84 | 0.287 |

Note: Bold words indicated p-values < 0.05.
